# Error in the Text

**DOI:** 10.1001/jamahealthforum.2026.0019

**Published:** 2026-02-20

In the Special Communication titled “Pharmacy Benefit Managers: History, Business Practices, Economics, and Policy,”^1^ published November 3, 2023, the first paragraph of the “Economics of the PBM Industry” section incorrectly stated that pharmacy benefit managers (PBMs) retained 9% (2012) to 22% (2016) of rebates in the commercial market. These percentages were swapped; the correct values are 22% for 2012 and 9% for 2016. This article has been corrected.^1^
